# Engaging Carers in Co-Design: Development of the Carer Readiness Tool

**DOI:** 10.5334/ijic.5527

**Published:** 2021-03-15

**Authors:** Sian White, Natalie Hart, Suzanne Lewis

**Affiliations:** 1 Clinical Safety, Quality and Governance Directorate, Central Coast Local Health District, Gosford, NSW, Australia

**Keywords:** informal caregivers, carer readiness, co-design, discharge planning, cancer patients

## Abstract

**Introduction:**

The Carer Support Unit (CSU) of the Central Coast Local Health District (CCLHD), NSW, Australia, developed, trialled and implemented a Carer Readiness Tool (CRT) to help carers gauge their readiness to care at home, highlight to hospital staff areas for additional support for carers, and provide evidence of carer engagement in discharge planning.

**Description:**

A rigorous co-design process was followed with carer consultation at key milestones in development of the CRT. The tool was piloted in two cancer/chronic renal disease inpatient units commencing November 2019.

**Discussion:**

The CRT was well-received by carers who appreciated the opportunity to complete the tool in their own time, not in front of the patient. Positive feedback was received from clinicians, including the breadth of the CRT’s content which contributed to better discharge planning. The need to manually incorporate a hard copy form into the electronic medical record is a limitation of the CRT.

**Conclusion:**

The CRT is context-specific and fit for purpose. During the development of the CRT, the project team focused on the face validity and usefulness of the tool. The next stage of the project will be formal evaluation of the tool to measure its impact.

## Introduction and Context

In recent years, delivery of health care has shifted away from hospitals and into the home, facilitated by improvements in treatment and technology and accompanied by increasing complexity in the care delivered in the home environment [1,2]. As a consequence, acute care services have increasingly collaborated with, and relied upon, informal caregivers. Recognising the importance of carers in the care pathway of cancer patients, the Carer Support Unit (CSU) of the Central Coast Local Health District (CCLHD) in NSW, Australia, worked with two inpatient cancer wards, one of which also delivers care to patients with chronic renal disease, to better support carers. The patients on these wards are usually admitted due to an acute exacerbation of an underlying chronic condition; while they have a primary diagnosis of either cancer or renal disease, most have a clinical profile of complex multi-morbidity. Given the typical patient cohort in these wards, it was anticipated that the majority of admitted patients would have a carer to support their needs at home.

The first stage of the project, a literature review and environmental scan to identify factors impacting the identification and recognition of carers, was carried out in 2019 [3]. The second stage of the project was to develop, trial and implement a Carer Readiness Tool (CRT). The purpose of the tool is threefold: most importantly, to help carers gauge their readiness and confidence to undertake caring tasks and responsibilities after the care recipient’s discharge from hospital; second, to indicate to clinical staff any areas in which the carer may need additional help, and help clinicians to gauge the limits of what carers may be able to do; and third, to provide evidence that CCLHD is engaging carers as partners in care. This paper reports the development and trial of the tool in late 2019.

## Ethical Approval

This project (Application ID: 2019/ETH12562) was approved under the low risk review pathway by the Human Research Ethics Committee, Hunter New England Local Health District, NSW, Australia.

## Literature Review

### Transfer of care into the community

A gradual transfer of care from hospital to home has taken place over the last twenty years or so, with the aim of integrating care pathways between acute and community settings, increasing patient choice, and supporting delivery of person-centred care. For this change to be successful, both patients and carers must be supported and confident to manage care at home. Dow and McDonald [4] identified the ‘invisible contract’ which assumes that much of the work of caring, previously delivered by health care professionals in an institutional setting, will instead be done by carers in the home. They challenged the assumption on which the model of care-at-home is based, that the home is a more therapeutic environment than the hospital. While people with complex, chronic conditions are generally treated in the community setting, they still require hospitalisation from time to time for acute exacerbations. Earlier discharge and increasingly technical forms of treatment at home, for example kidney dialysis, mean that the successful transfer of care for patients with complex, chronic conditions from the acute hospital environment to the community has come at a substantial and largely hidden cost to carers [1,4].

Olsen’s qualitative study of 32 carers of spouses with cancer proposes that not only the location of care, but also the responsibility for coordination of care, has shifted to informal caregivers. Carers described undertaking a care coordination role akin to project management. They organised appointments, followed up referrals, and kept records of consultations, scans and prescriptions, in addition to providing personal care, delivering medical and nursing interventions, and providing emotional support to their spouse [5].

### Discharge planning

There is strong evidence that early and thorough consultation between hospital staff and carers results in fewer readmissions to hospital [6]. Despite this evidence, confusion, poor communication and lack of planning around discharge from hospital have been identified as pressure points for carers [4]. Carer experiences after discharge of the person they care for from hospital are characterised by feelings of uncertainty [2,7]. Perceived lack of discharge preparation and information resulted in carers and patients not knowing what was ‘normal’, leading to either over- or under-vigilance [2].

Aoun and colleagues identified communication failures between hospital staff and caregivers at discharge, arising from both lack of time and lack of opportunity, as a significant problem, ‘compounded by the lack of a suitable assessment tool for caregivers’ needs that can be used routinely and systematically by hospital staff’. Written evidence of carer involvement in discharge planning is limited, fragmented, discipline-specific and often buried in progress notes in the patient’s medical record [8, p.1].

### Medicalisation of the Home

Medication management and information about dosage and possible side effects have been flagged by carers as areas of significant concern [9]. Medical or nursing tasks previously performed by a health professional in hospital pose major challenges for carers, and contribute to feelings of unpreparedness [1,10].

Reinhard and colleagues’ ‘Home Alone Revisited’ study, based on the online survey responses of 2,089 family caregivers providing care to someone with a chronic physical or behavioural condition, disability or addiction, found that ‘[t]oday’s caregivers provide intense and complex care, including performing medical/nursing tasks and managing multiple health conditions that are often accompanied by pain’ [1, p.i]. The range of tasks being performed by carers included medication management, pain management, help with mobility, special diets, wound care, operating monitors and other medical equipment, incontinence management including catheters, supplementary oxygen delivery, intravenous medications, tube feeding, suctioning, home dialysis and ostomy care [1, p.9]. The authors urge ‘[h]ealth care systems and professionals….[to]make stronger efforts to recognize family caregivers and offer them instruction on and support for complex care’ [1, p.iii].

### Carer Readiness for Care

Dealing with the multiple demands of caregiving requires knowledge, skills, problem-solving ability and judgement [7]. Caregivers are often facing a triple burden of care: personal care of the patient such as toileting, showering and dressing; household care including shopping, cleaning, finances; and responsibility for ongoing medical/nursing tasks in the home. Carers may also have competing demands on their time and attention such as work and care of children or elderly parents. They may or may not have the support of other family members, neighbours, community groups or formal support services, and may be under financial stress [7]. All these factors can determine the preparedness of the carer to undertake care, but how can their preparedness or readiness be assessed in a busy acute inpatient ward?

Ewing and colleagues’ qualitative study of carer support during hospital discharge of patients at end of life found that comprehensive assessment of carer needs is unlikely to take place in most hospital settings due to organisational focus on the needs of the patient, and high staff workload. As one healthcare practitioner commented, ‘“Honestly the carers are my second thought and they are only my thought if there becomes a difficulty [with discharge]”’ [11, p.942]. Other factors can make it difficult for carers to discuss their readiness to provide medical, nursing, personal or household care with hospital staff. They may be reluctant to express their concerns in front of the patient; they may not want to ‘interrupt’ busy clinicians; they may be confused about clinical roles; or may not feel comfortable focusing on their own needs instead of those of the patient [3].

## Development of the Carer Readiness Tool

The CCLHD CSU project team aimed to introduce a carer capacity assessment tool into the workflow of the two inpatient acute cancer/renal wards. The tool had to be easily understood by carers and quick to complete. Development had to be carer-driven, in line with NSW Health policy requirements to consult carers in the development of any documentation relevant to them, and in service planning, delivery and review processes [12]. Finally, the tool had to bring the carer voice into the discharge planning process, making their needs ‘visible, legitimised and acknowledged’ [8, p.8].

### Phase One: Review of existing tools

Several pre-existing tools were identified and assessed to determine whether they would be suitable for the purpose and context of this project.

The Carer Support Needs Assessment Tool (CSNAT) is the most well-known tool used to assess carers’ support needs. Its focus is on identifying global need and the overall burden of care in the context of palliative and end-of-life care. Health service organisations are required to commit to the CSNAT approach, including online training which must be completed before a licence can be issued to use the CSNAT checklist. The checklist itself is copyrighted and cannot be altered in any way [13]. The project team and clinical staff felt that it was not feasible to implement the CSNAT approach in the acute setting of inpatient cancer units.

The Needs Assessment Tool for Carers (NAT-C) [14] is a checklist for carers of people with advanced cancer, designed to be used in consultations with their general practitioner. The 33 items in the checklist are organised into the following five domains: information issues, practical issues, personal health and well-being issues, relationship issues and meaning issues [15]. While these all have the potential to impact on a carer’s ability to care for the patient post-discharge, it was felt that a busy acute, inpatient unit was not the appropriate setting in which to expect carers to explore relationship and meaning issues.

The Family Caregiver Activation in Transitions (FCAT) Tool [16,17] was developed to measure caregiver self-efficacy during transitions between healthcare providers and settings, including discharge from hospital to home. The 10-item tool requires the carer to self-rate their carer effectiveness, specifically, their knowledge, capacity, organisational ability and personal qualities.

The pre-existing tools we reviewed focus on measuring carer self-efficacy, quality of life and the burden of care. Our focus was on carer *readiness* to undertake care at home, and while self-efficacy and quality of life are important related concepts, none of the tools described above were considered to be ideal for our purpose. We required a tool that could be completed independently by the carer any time after the patient’s admission to the ward and before discharge. It would assist carers to identify aspects of care they may not have considered previously, and would be prospective, rather than retrospective, focusing on the current situation and immediate future. We also wished to avoid the judgement implied in asking carers to self-rate or assess their capacity to provide care.

The Next Step in Care project of the US-based United Hospital Fund provides a suite of guides and checklists for family caregivers. The checklist “What do I need as a Family Caregiver” [18] was identified as containing most of the elements required for a Carer Readiness Tool for use by carers of patients in the CCLHD inpatient cancer wards.

### Phase Two: Development of a draft CRT and cognitive testing

The project team, consisting of the CSU Manager, CSU staff and an occupational therapist recruited as project officer, commenced a process of successive drafts of the CRT. Carers were actively involved in the co-design process throughout. A purposive sample of carers was recruited via direct approach from either the project team or the multidisciplinary clinical staff on the wards. Carers of medically unstable patients or patients judged to be at the end of life were not approached to participate; nor were carers relinquishing their caring responsibilities at home, for example, by admission of the care recipient into residential aged care. Initially, 14 carers took part in one-hour interviews with CSU staff, working through successive iterations of the tool which, at this stage, consisted largely of open-ended questions. Thematic analysis was applied to their responses until saturation was reached.

The team then returned to the existing tools identified in the literature search to guide the process of distilling a one-hour interview with open-ended questions into a checklist able to be completed in ten minutes. The team decided to avoid the use of Likert scales and scores, which infer judgement of carer capacity. For this reason, the initial name of the tool, the Carer Capacity Assessment Tool, was changed in later drafts to the Carer Readiness Tool.

The second iteration of testing was carried out during 30-minute interviews with a further 15 carers, recruited in the same way as the previous group. At this stage the tool consisted of a combination of checklist items plus open-ended questions. This stage was used to identify which items were useful and which should be discarded; and determined whether each checklist item was readily understood by carers.

Once the CRT reached its final draft format, a third group of 18 carers completed the checklist during an interview with CSU staff. The demographic profile of the 47 carers consulted is shown in ***Table 1***.

**Table 1:** Demographic characteristics of carers consulted.


AGE	

18–35 years	4 (8.5%)

36–64 years	15 (32%)

65+ years	28 (59.5%)

**GENDER**	

Female	29 (62%)

Male	18 (38%)

**CALD (CULTURALLY AND LINGUISTICALLY DIVERSE)**	**6 (13%)**

**ATSI (ABORIGINAL OR TORRES STRAIT ISLANDER)**	**3 (6%)**

**EMPLOYMENT STATUS**	

Employed	7 (15%)

Retired	27 (57%)

Unemployed (and less than 65 years)	13 (28%)

**TIME IN CARING ROLE**	

Less than 1 year	17 (36%)

1–5 years	14 (30%)

More than 5 years	12 (25.5%)

Unspecified (respondents did not answer/were unsure)	4 (8.5%)

**CARERS IDENTIFYING THEIR OWN HEALTH ISSUES**	**15 (32%)**


During all three stages of development, an expert panel of four carers provided online feedback at inception and at key change points in the tool’s development. This core group has worked with CSU staff for some years and volunteered for this and other projects as a sounding board for issues and challenges.

The project team contacted the United Hospital Fund and received permission to use the “What do I Need as a Family Caregiver” checklist and adapt as necessary. The elements of the “What do I Need…?” checklist judged to be most relevant and useful for modification and inclusion in the CRT were reordered and renamed for the CRT as shown in ***Table 2***.

**Table 2:** Adaptation of content from the “What do I Need as a Family Caregiver?” checklist to the CCLHD Carer Readiness Tool.


WHAT DO I NEED AS A FAMILY CAREGIVER (UNITED HOSPITAL FUND 2008)	CARER READINESS TOOL (CCLHD)

Section 1 – “About You as the Family Caregiver” (employment, living arrangements, other caring responsibilities and general health) Section 3 – “About Services at Home/Community” (home care services currently in place and additional services that may be required).	Section 1 – What do you need as a Carer?

Section 2 – “About Helping Your Family Member” (ability to help with personal care of the patient, medication management, management of medical devices, transport, coordinating care, financial management and more)	Section 2 – Before the patient goes home what help do you need from us?

Section 4 – “About Worries” (areas of concern)	Section 3 – What concerns do you have?


### Phase Three: Pilot testing

A final pilot version of the CRT was prepared and progressed through health service governance processes to arrive at an endorsed CRT trial form, ready for introduction on the wards (See Appendix 1: Carer Readiness Tool, Trial Form October 2019).

Immediate benefits of the co-design process have been identified. Carers reported their appreciation of being formally asked about their needs and concerns.

“It was refreshing to actually be asked and then have the opportunity to write down my concerns in my own time.”

“I think the questionnaire is a great idea. When my husband was first diagnosed I felt I was a number in a system. Anything that made me feel ‘seen’ and individual was appreciated”.

Carers were able to document competing caring demands, existing formal and informal supports, and any concerns they had without having to express these in front of the patient.

“I was really frightened about him dying at home, once that was out in the open the social worker had a quiet chat with me about this. It made all the difference.”

Just under half of the carers who participated in the co-design of the CRT were connected by the CSU to emotional, financial and/or practical supports during or after the project.

During the CRT development process, the project team was also working with staff of the two cancer/renal inpatient units. The team spent 88 hours on each ward over a period of 26 weeks (a total of 176 hours) providing feedback from carers to clinical staff and troubleshooting the introduction of the CRT on the wards. A number of challenges were encountered during early implementation of the CRT. The form is given to carers to complete in hard copy and incorporation of the information into an electronic patient record presents logistical challenges. Change fatigue on the part of both staff and carers – “not another form” – is also an issue.

Positive feedback from clinicians included comments regarding the breadth of the CRT’s content as well as the fact that it was clear evidence that consultation had occurred with the carer.

“[T]o be honest, some of the questions are things that I had not thought to ask of carers”. (Social Worker)

“I can see the value in checking in with carers regarding each indicator – carers may not voice concerns if they are not asked about specific issues.” (Social Worker)

“Often patients report very different information than their family members/carers”. (Nurse in Charge, Oncology Ward)

The CRT incorporated into the patient’s medical record provides written evidence of carer consultation, which is a requirement for hospital accreditation as measured against the Australian National Safety and Quality Health Service (NSQHS) Standards [19]. It is also evidence of compliance with the *NSW Health Recognition and Support for Carers: Key Directions 2018–2020* guideline [12].

**Figure 1:**
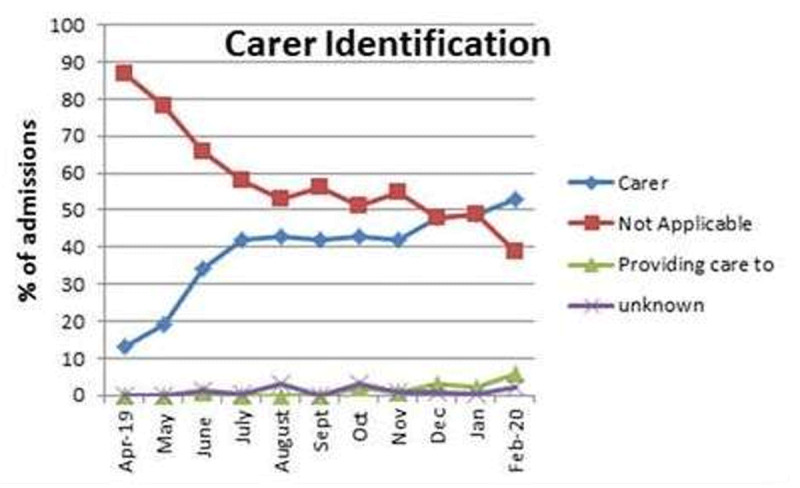
Rate of carer identification in the eMR for patients admitted to the two cancer/renal inpatient wards, April 2019 to February 2020.

Since the project began in early 2019, the rate of carer identification in the electronic medical record (eMR) for these two wards has increased from a baseline of 13% of all admitted patients’ records in April 2019 to 42% in November 2019 when the CRT was introduced for use on the cancer/renal wards, to 53% in February 2020 is shown in ***Figure 1***. Use of the CRT appears to be addressing one of the major concerns identified early in the project, that carers were not being identified correctly during patient admission, and were not correctly identified in the eMR [3].

## Discussion

The development of the Carer Readiness Tool was driven by carers’ identification of their concerns during a rigorous, iterative process of testing and modification. This process ensured that each item is readily understood, and the CRT can be completed quickly if need be. Some of the pre-existing tools identified by the project team ask carers to reflect retrospectively on their caring experience. The CRT, however, focuses on the ‘here and now’ and immediate requirements of caring for a patient about to be discharged from hospital. It is a prompt to carers to consider issues they may not have thought of previously, and an indicator to clinical staff of where additional support may be needed. Carers are not asked to rate their own ability to perform certain tasks. Instead they are asked to indicate which caring tasks they may need help with by selecting from the following options: Do not need help; Need help; Unsure, please talk with me.

The need to manually incorporate a hard copy form into the largely electronic medical record is a limitation of the CRT. It is also acknowledged that carer capacity to care is built over time and is not a static concept. The CRT provides a snapshot of readiness to care which reflects the current situation but does not necessarily predict future circumstances and capacity [20]. It is envisaged that the tool will be adapted for use depending on the operational requirements of each ward in which it is deployed. However regardless of context, the CRT will support better communication between clinical staff and carers, and inform discharge planning to ensure a smooth transition of care from hospital to home, with less likelihood of an unplanned readmission.

During the development of the CRT, the project team focused on the face validity and usefulness of the tool. The next stage of the project will be formal evaluation of the tool to measure its impact. Eligible carers will be offered the CRT for completion and invited to participate in a follow-up telephone interview to explore the impact of the tool using the Preparedness for Caregiving Scale [21,22].

## Conclusion

The CCLHD Carer Readiness Tool was developed using a rigorous co-design approach with carers driving each stage of the process. The result is a tool fit for both purpose and context which meets the three goals identified early in the project: it helps carers identify what help they may need to be ready to care at home; it flags to clinical staff any additional help that a carer may need prior to patient discharge; and it is clear evidence that CCLHD is engaging carers as partners in care. The CRT has the potential to improve care integration by ensuring that the carer voice is heard, and informs clinical decisions around care and discharge planning. Carers who are recognised, well-prepared and supported are essential to ensuring better outcomes for patients, particularly those with chronic, complex conditions and multi-morbidity.

## Additional File

The additional file for this article can be found as follows:

10.5334/ijic.5527.s1 Appendix 1. Carer Readiness Tool.
