# Supplementary material for: Engaging Carers in Co-Design: Development of the Carer Readiness Tool
Source: Int J Integr Care. 2021 Mar 15;21(1):13. doi: 10.5334/ijic.5527 (PMC7977025; doi:10.5334/ijic.5527)
Supplement: Appendix 1. — Carer Readiness Tool. [file ijic-21-1-5527-s1.pdf]

Health
Central Coast
Local Health District

Dear Carer

Your family member/friend has identified you as someone they rely on for regular help at home. The Central Coast Local Health District recognises you as a Carer and is inviting you to complete the Carer Readiness Tool.

The tool asks you to write down any concerns you may have in relation to your caring role. Completion of this form is important because it will help us make sure that you and your family member/friend are ready to go home from hospital and if needed you are linked in with services once you get home.

After you have completed the tool your answers will be discussed with the ward-based team of health workers, and referrals may be made to different workers on the ward or to the Carer Support Unit for help.

If staff are not able to talk with you on the ward they may contact you by phone. When staff from the hospital phone it will be from a private or blocked number. Please do not be alarmed.

If you need help with the form please ask for the Allied Health Assistant or call the Carer Support Unit on 4320 5556.

Thank you

Carer Support Unit and Nursing Unit Manager

CATALOGUE NUMBER CC 12249 JUL20/V2

CCLHD CONSUMER FRIENDLY RESOURCE

Central Coast Local Health District
ABN 88 523 389 096
Gosford Hospital
PO Box 361 Gosford NSW 2250
Tel. (02) 4320 2111
www.cclhd.health.nsw.gov.au

Health
Central Coast
Local Health District

Facility: COM GOS LJ WW WYG

CARER READINESS TOOL

FAMILY NAME	MRN
GIVEN NAME	☐ MALE ☐ FEMALE
D.O.B. DD / MM / YYYY	M.O.
ADDRESS	
	PH
M/C	FIN
LOCATION / WARD	ADM DD / MM / YYYY

COMPLETE ALL DETAILS OR AFFIX PATIENT LABEL HERE

AST5333

Staff Name .... Designation ....

Date Completed: __/__/____

About You

Name .... Relationship to Patient ....

Contact Number .... Best time to contact ....

What do you need as a Carer?

How long have you been involved in the patient's care?

☐ I haven't, this is new (patient was caring for self)

☐ I've been doing this for a while. How long? ....

How easy is the patient's home for you to reach?

☐ Same house/unit

☐ Easy to get to

☐ Not easy/a long trip

☐ I really can't (out of state/country or other reason)

Do you work/study/volunteer? ☐ Yes ☐ No

If yes, are you: ☐ Full time ☐ Part Time

Are you raising children under the age of 18? ☐ Yes ☐ No

Are you also a carer for someone else with health problems or a disability? ☐ Yes ☐ No

Do you have any health problems that affect you as a Carer? ☐ Yes ☐ No
(e.g. back problems, arthritis, diabetes)

If yes, please list here:

....

....

....

Will other people help with care? (e.g. family members or friends) ☐ Yes ☐ No

Are there other services involved? (check all that apply)

☐ Adult Day Care ☐ Home Care ☐ Home Companion ☐ NDIS

☐ Meals on Wheels ☐ Transport ☐ My Aged Care Services

Do you have any other information or questions at this time?

....

....

....

....

....

....

Holes punched as per AS2828 - 2012

BINDING MARGIN – NO WRITING

CATALOGUE NUMBER CC 12249 JUL20/V2

CARER READINESS TOOL - CARER SUPPORT

MARGIN – NO WRITING

NSW GOVERNMENT | Health
Central Coast
Local Health District

Facility: COM GOS LJ WW WYG

CARER READINESS TOOL

FAMILY NAME		MRN
GIVEN NAME		☐ MALE ☐ FEMALE
D.O.B. DD / MM / YYYY	M.O.	
ADDRESS		
		PH
M/C	FIN	
LOCATION / WARD		ADM DD / MM / YYYY

COMPLETE ALL DETAILS OR AFFIX PATIENT LABEL HERE

Before the patient goes home, to be prepared, what help do you need from us?

	Do not need help	Need help	Unsure, please talk with me
1. Washing (shower, bath or bed)	☐	☐	☐
2. Dressing (getting dressed or undressed)	☐	☐	☐
3. Eating and swallowing	☐	☐	☐
4. Grooming (brushing teeth, washing hair and cutting nails)	☐	☐	☐
5. Toileting (going to the bathroom or continence support)	☐	☐	☐
6. Transfer (from bed to chair, chair to toilet or wheelchair to car)	☐	☐	☐
7. Walking or moving about	☐	☐	☐
8. Aids and equipment (such as oxygen or shower bath chair)	☐	☐	☐
9. Strategies to support the person's behaviour and/or feelings	☐	☐	☐
10. Medication (buying and giving medication)	☐	☐	☐
11. Looking after symptoms (such as pain and nausea)	☐	☐	☐
12. Coordinating care (talking with doctors, nurses, and other health care workers)	☐	☐	☐
13. Making and keeping appointments	☐	☐	☐
14. Driving or helping with transport (such as car, bus or taxi)	☐	☐	☐
15. Household tasks (such as shopping, cooking, cleaning and doing laundry)	☐	☐	☐
16. Taking care of finances (including banking, paying bills, forms and applications)	☐	☐	☐
17. Information about parking at the hospital	☐	☐	☐

What concerns do you have?

- ☐ Your level of stress and how to cope with it
- ☐ How to balance work/family/study/volunteering and your caring role
- ☐ How to get time off (respite from being a carer)
- ☐ What the person's health condition means to you and others who care about him or her
- ☐ How to deal with your own strong emotions
- ☐ How to look after your own health and wellbeing in addition to your caring role
- ☐ Whether the person is safe at home, or what to do if he or she wanders
- ☐ Where the person lives, and if this needs to change (such as moving to a nursing home or assisted living)
- ☐ Making health care decisions on behalf of the person your care for (being the Person Responsible)
- ☐ Legal issues (such as Advance Care Planning, Enduring Guardianship, Power of Attorney and other paperwork)
- ☐ How to pay for care/medications/services
- ☐ How to talk about what is going on with family and/or friends
- ☐ How to manage medications and perform (medical) care tasks
- ☐ What to do if the person you care for needs end-of-life care
- ☐ Knowing what to do in an emergency (change in health/fall)

Other concerns?

....

....

©2011 United Hospital Fund. Adapted with permission

BINDING MARGIN – NO WRITING

Holes punched as per AS2828 - 2012
